# The Mickey Mouse Sign: A Novel Sign for a Compliant and Underactive Bladder Syndrome

**DOI:** 10.7759/cureus.72702

**Published:** 2024-10-30

**Authors:** Faris Abushamma, Rola Abu Alwafa, Safaa Abatli, Maha Akkawi, Abdelkarim Barqawi

**Affiliations:** 1 Department of Medicine, College of Medicine and Health Sciences, An-Najah National University, Nablus, PSE; 2 Department of Urology, An-Najah National University Hospital, Nablus, PSE; 3 Department of General Surgery, An-Najah National University Hospital, Nablus, PSE; 4 Department of Pathology, An-Najah National University Hospital, Nablus, PSE

**Keywords:** intermittent self-catheterization (isc), mickey mouse sign, underactive bladder, urinary symptoms, urodynamics

## Abstract

This article represents a unique CT-based sign called the Mickey Mouse sign in a diabetic female who presented with abdominal distension and mixed urinary symptoms. The CT scan showed that the bladder was massively dilated but compliant without impacting the upper urinary tract. The subtle clinical presentation and the impressive CT scan findings encouraged us to report this case as underactive bladder syndrome (UAB) is underreported and underinvestigated. Furthermore, the management and clinical course of UAB in such cases remain controversial.

## Introduction

Underactive bladder syndrome (UAB) is a challenging clinical syndrome that poses a diagnostic and therapeutic dilemma. The International Continence Society recently recognized UAB as a separate clinical entity to further identify its causes and help in its management. The main clinical features of UAB are subtle, characterized by slow urinary stream, hesitancy, and straining to void, with or without a feeling of incomplete bladder emptying [1-6]. Thus, there is no evidence in the literature to support how much the bladder should be accommodative and whether the high residual volume inside the bladder may cause harm to the kidneys in UAB. Moreover, the underlying cause of UAB is not always obvious, as, in many cases, UAB may be the initial presentation of a serious underlying disease such as cancer and spinal cord compression [7]. Invasive urodynamics is the standard method to evaluate UAB according to the European Association of Urology and American Urological Association guidelines. During urodynamics, the voiding phase shows detrusor underactivity (DUA), which is a contraction of reduced strength and/or duration, resulting in failure or prolonged bladder emptying. Diabetes mellitus (DM) is a common cause of UAB as more than half of the patients with longstanding and poorly controlled DM tend to develop diabetic bladder dysfunction [8]. However, it is rarely reported that DM-related UAB may cause massive bladder distention reaching up to the kidney without causing significant hydronephrosis [9]. Thus, we report the case of a 64-year-old female with poorly controlled DM who presented with mixed lower urinary tract symptoms (LUTS) and abdominal distention. We found on her CT scan what we call the *Mickey Mouse sign* which represents a massively dilated and compliant bladder.

## Case presentation

**Figure 1:**
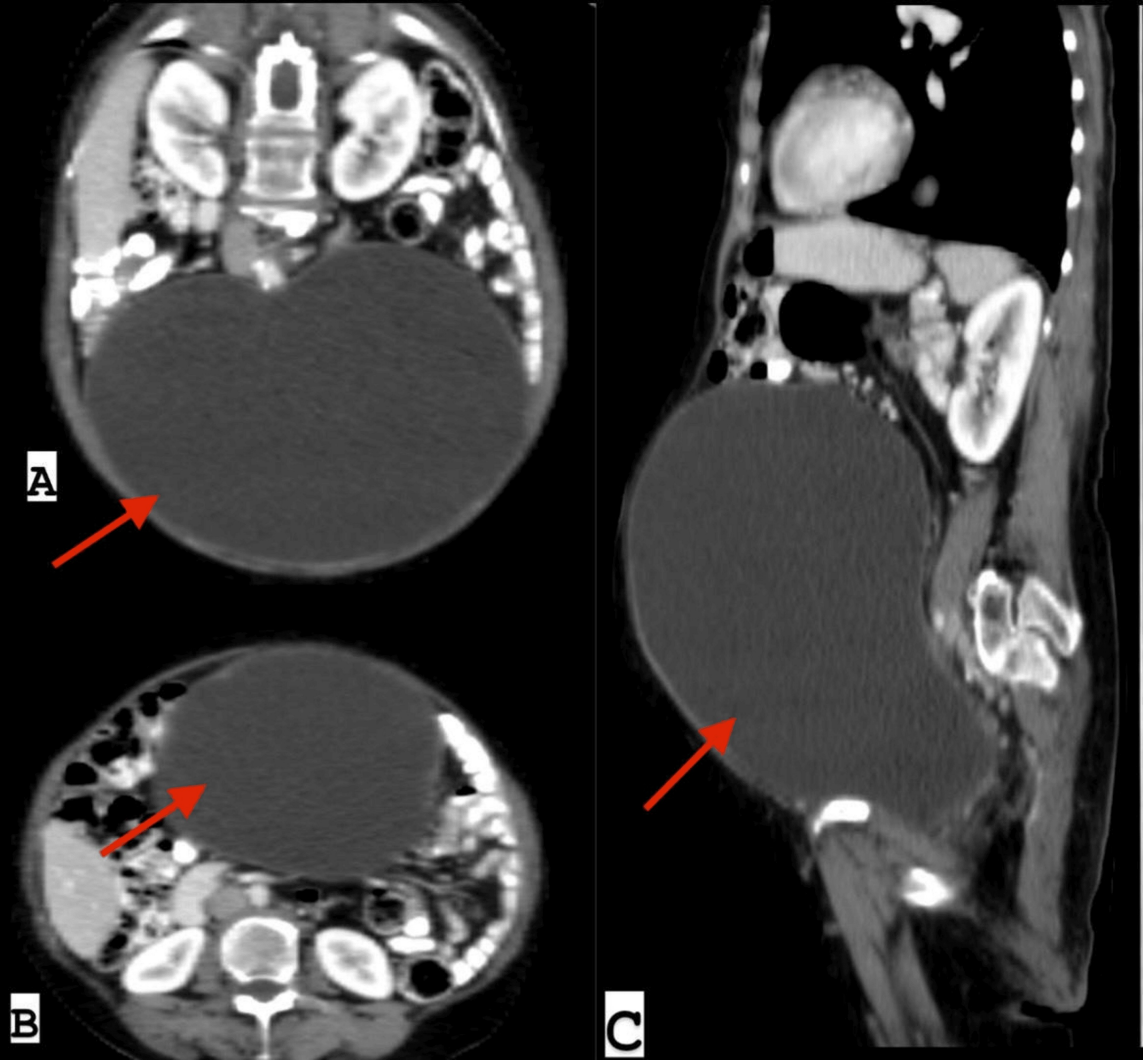
CT scan findings. A: Coronal oblique CT scan with IV and oral contrast showing hugely distended urinary bladder, reaching the level of kidneys. The bladder wall is smooth without irregularities. B: Axial CT scan with IV and oral contrast showing hugely distended urinary bladder, compressing other abdominal organs. C: Sagittal CT scan with IV and oral contrast showing the bladder replacing and compressing the small bowel, reaching up to the stomach level.

A 64-year-old female with poorly controlled DM presented with abdominal distension and mixed LUTS for a few months. Her LUTS included both storage symptoms, such as frequency and urgency, and voiding symptoms, such as a weak stream and straining with urination. She also reported a history of recurrent, subtle urinary tract infections treated with oral antibiotics for more than one year without proper evaluation or documented previous urine cultures. There was no history of gross hematuria or sepsis. Due to her slim build and a body mass index of 19 kg/m^2^, the abdominal distension was clinically evident. A CT scan with IV and oral contrast was performed to investigate the cause of the abdominal distension, revealing a significantly distended urinary bladder extending to the level of both kidneys and compressing the abdominal organs. Both kidneys showed no hydronephrosis and retained good renal parenchyma (Figure 1).

**Figure 2:**
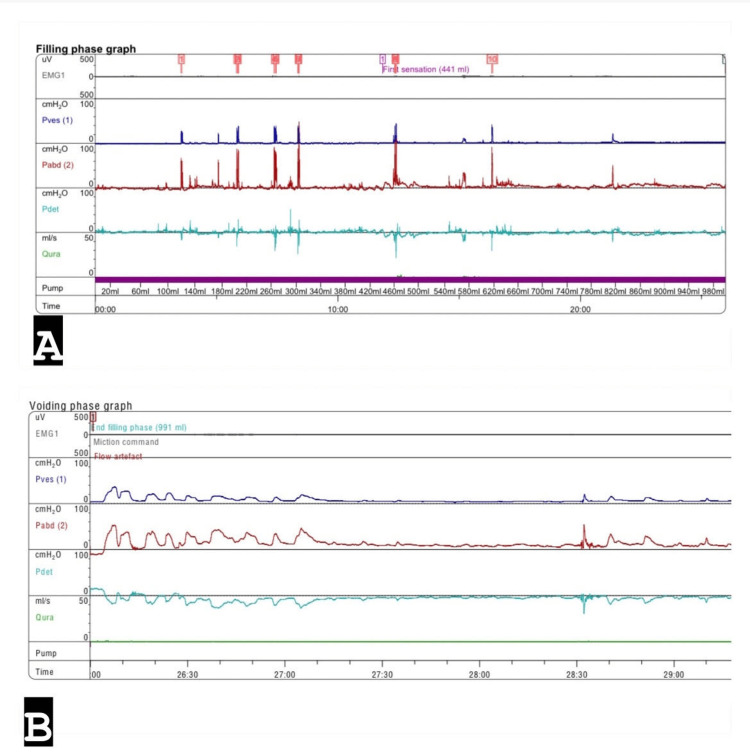
Urodynamic findings. A: The filling phase of the urodynamic study shows good compliance and no detrusor overactivity. The cytometric capacity is 1,000 mL. B: The voiding phase of the urodynamic study shows abdominal contraction with no flow and no evidence of detrusor function.

Following these findings, a Foley catheter was inserted, draining approximately 6-7 L of urine. This volume of urine indicated bladder dysfunction. A multidisciplinary uro-radiology team determined that the case likely represented UAB, warranting further investigations through invasive urodynamics. Invasive urodynamics was performed in a semi-sitting position. During the filling phase, the complete absence of bladder sensation led to the cessation of infusion at 1,000 mL, which was considered the maximum cystometric capacity. Bladder compliance was within normal limits, and the detrusor pressure during filling (Pdet) showed no signs of detrusor overactivity. No urine leakage was observed during the maximum Valsalva leak test (Figure 2A), illustrating the filling phase of the invasive urodynamics. During the voiding phase, the patient attempted to use her abdominal muscles without any evident Pdet contractions (Figure 2B), indicating her abdominal effort to void without a rise in Pdet.

The urodynamic diagnosis confirmed DUA secondary to poorly controlled diabetes. Her HbA1c level was 13 which indicated poor glycemic control. Based on these findings, We recommended initiating intermittent self-catheterization (ISC) and tighter diabetes management. After 18 months of close monitoring, despite strict diabetes control and the use of ISC, there was no significant improvement in her UAB. Repeated urodynamics showed no enhancement in the voiding phase with no detectable detrusor contractions. A three-month trial of Myocholine-Glenwood 25 mg twice daily also failed to alleviate her clinical symptoms. Consequently, her long-term options are either ongoing catheterization or continued ISC.

## Discussion

**Figure 3:**
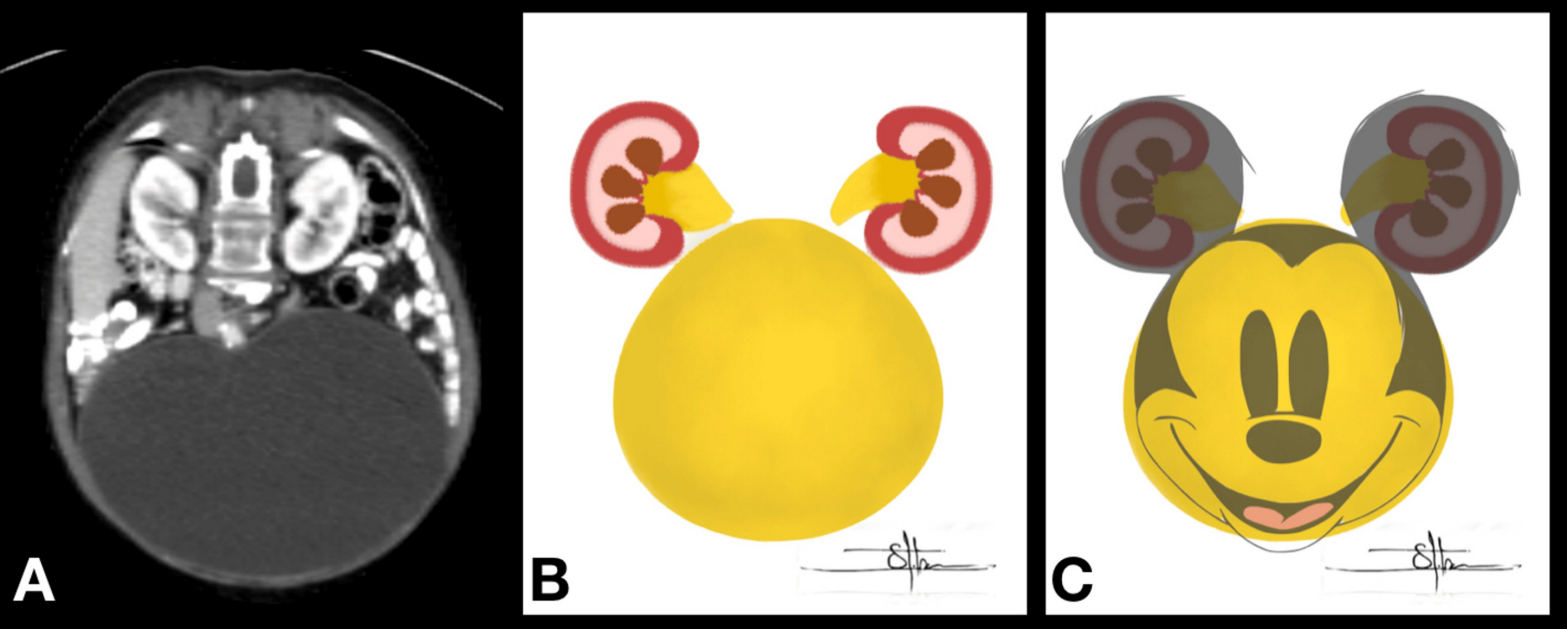
Demonstration of the radiological sign. A-C: The diagram illustrates the formation of the *Mickey Mouse sign*, where we believe the significantly enlarged bladder represents the face, and the undilated kidneys act as the ears. The image is original, authentic, and drawn by the co-author, SA.

The *Mickey Mouse sign* is a radiological sign previously described in the bladder containing bilateral inguinal hernias on CT scans and bone scintigraphy in patients with Paget’s disease [9,10]. In our case, this sign was characterized by significant smooth bladder distension reaching the lower poles of both kidneys, while remarkably not inducing substantial hydronephrosis in either kidney (Figure 3; an original image drawn by the co-author (SA) with a funny demonstration of such a radiological sign to make it memorable among medical students and urology trainees). The *Mickey Mouse sign* is a descriptive term rather than a diagnostic one, as this sign has been observed in various disease entities. Its use in our case is to aid in the visualization and description of the radiological findings rather than to imply a specific diagnosis.

A distinctive aspect of this case is that bladder distension in UAB can lead to significant abdominal enlargement, pressing against other organs and filling the entire abdominal cavity, yet without exerting pressure on the kidneys, which diverges from earlier reports [11]. In this instance of poorly controlled DM, the patient exhibited bladder distention with good compliance, resulting in a protective effect on the kidneys despite the large residual urine volume. This characteristic contrasts with cases of poorly compliant bladders, where increased residual urine volume can potentially cause kidney damage [9]. The management of such a case is based on surgeon experience as UAB has received a lack of attention regarding understanding its pathophysiology and management over many years [12]. For instance, ISC, sacral neuromodulation, and off-label medications such as Bethanechol are all potential treatment options, albeit, the outcome might not be optimal, as in our case. To sum up, this case represents a unique presentation of UAB manifested by the massive residual volume which did not affect the upper tract despite the bladder replacing the abdominal content toward the lower poles of both kidneys. Such a unique CT, which captured the interest of our trainees, radiologists, and medical students, is known as the *Mickey Mouse sign*, contrasting with the *Christmas tree sign* used to depict the look of a hypertonic, neurogenic bladder.

## Conclusions

The identification of the *Mickey Mouse sign* in CT imaging as a marker for UAB offers a valuable descriptive tool in medical diagnostics. This unique imaging feature, observed in our case, demonstrates a compliant bladder capable of accommodating a massive residual volume without adversely impacting the upper urinary tract. While this finding is notable, it is important to acknowledge that it is based on a single case, and further research is needed to validate its diagnostic utility across a broader population. Given the complexity of UAB, the identification of such distinctive imaging features can aid clinicians in recognizing and diagnosing this condition earlier and more accurately. This case points to the potential for specific radiological signs to enhance diagnostic precision and contribute to improved patient management, but it should be interpreted with caution until supported by larger studies. The *Mickey Mouse sign*, as observed in this patient, highlights the importance of integrating detailed imaging analysis into the comprehensive evaluation of UAB, ultimately aiming for better patient outcomes through timely and appropriate interventions.
